# Human Endogenous Retrovirus-K(II) Envelope Induction Protects Neurons during HIV/AIDS

**DOI:** 10.1371/journal.pone.0097984

**Published:** 2014-07-02

**Authors:** Rakesh K. Bhat, Wallis Rudnick, Joseph M. Antony, Ferdinand Maingat, Kristofor K. Ellestad, Blaise M. Wheatley, Ralf R. Tönjes, Christopher Power

**Affiliations:** 1 Department of Medicine (Neurology), University of Alberta, Edmonton, Alberta, Canada; 2 Department of Surgery (Neurosurgery), University of Alberta, Edmonton, Alberta, Canada; 3 Division of Medical Biotechnology, Paul-Ehrlich-Institut, Langen, Germany; Queen’s University, Canada

## Abstract

Human endogenous retroviruses (HERVs) are differentially expressed depending on the cell type and physiological circumstances. HERV-K has been implicated in the pathogenesis of several diseases although the functional consequences of its expression remain unknown. Human immunodeficiency virus (HIV) infection causes neuroinflammation with neuronal damage and death. Herein, we investigated HERV-K(II)/(HML-2) envelope (Env) expression and its actions in the brain during HIV/AIDS. HERV-K(II) Env expression was assessed in healthy brain tissues, autopsied HIV HIV− infected (HIV+) and uninfected (HIV−) brains and in neural cell cultures by real time RT-PCR, massively parallel (deep) sequencing, immunoblotting and immunohistochemistry. Neuronal and neural stem cells expressing HERV-K(II) Env were analyzed in assays of host responses including cellular viability, immune responses and neurobehavioral outcomes. Deep sequencing of human brain transcriptomes disclosed that RNA sequences encoded by HERV-K were among the most abundant HERV sequences detected in human brain. Comparison of different cell types revealed that HERV-K(II) *env* RNA abundance was highest in cultured human neurons but was suppressed by epidermal growth factor exposure. HERV-K(II) Env immunoreactivity was increased in the cerebral cortex from persons with HIV/AIDS, principally localized in neurons. Human neuronal cells transfected with HERV-K(II) Env exhibited increased *NGF* and *BDNF* expression. Expression of HERV-K(II) Env in neuronal cells increased cellular viability and prevented neurotoxicity mediated by HIV-1 Vpr. Intracerebral delivery of HERV-K(II) Env expressed by neural stem cells suppressed TNF-α expression and microglial activation while also improving neurobehavioral deficits in *vpr/RAG1^−/−^* mice. HERV-K(II) Env was highly expressed in human neurons, especially during HIV/AIDS, but in addition exerted neuroprotective effects. These findings imply that HERV gene products might exert adaptive effects in circumstances of pathophysiological stress, perhaps underlying the conservation of HERVs within the human genome.

## Background

Human endogenous retroviruses (HERVs) represent approximately 8% of the human genome, which have been maintained through integration events over the past 50–100 million years [1], [2], [3]. In humans, endogenous retroviruses are not replication competent but can be engineered to replicate productively [4]. Endogenous retrovirus genes are inherited in a Mendelian manner in different species, usually remaining latent, but can become active depending on the individual cell type and host health status [5]. Although the human genome harbors a large number of endogenous retroviral sequences, their action(s) remain largely uncertain at present. We have shown previously that the human endogenous retrovirus (HERV)-W envelope protein, Syncytin-1, is highly expressed in glial cells within brain lesions of patients with multiple sclerosis and also contributes to endoplasmic reticulum stress [6], [7].

HERV-K represents the most recent entry into the human genome and is also detected as multiple sub-types in humans [8]. There have been several disease associations with HERV-K [9], [10], [11], [12]. The beta-retroviral HERV-K (HML-2), also referred to as the HERV-K(II) family, is considered to be among the youngest member of the HERVs and exhibits multiple polymorphic insertions, indicative of recent active replication in humans [8], [13], [14]. We previously showed that HERV-K(II) is one of the most transcriptionally active HERV families in brain and might be capable of generating virus-like particles [15]. Abnormal expression of HERV-K(II) proteins or transcripts has been associated with different pathological circumstances [16], [17]. For example, induction of HERV-K *pol* transcript expression was reported in post-mortem brains from individuals with schizophrenia and other neuropsychiatric disorders [18], [19], [20]. HERV-K gene activation also occurs in different cancer cell lines and tumors [21]. Our group has previously shown an augmented expression of HERV-K *pol* transcripts in the brains of patients with neuroinflammatory disorders [22]. The high HERV-K Env amino terminal sequence conservation with Jaagsiekte sheep retrovirus (JSRV), which is contagious and causes lung cancer in sheep, suggests that the HERV-K Env might share similar properties in terms of receptor binding or modulating cellular entry [23], [24]. However, it remains unclear if HERV-K genes exert pathogenic (or protective) effects.

During HIV/AIDS, HERV-K is highly expressed in blood although the determinants of its transcription and translation remain unclear [25], [26]. Whether the increased expression of HERV-K in persons with HIV/AIDS requires specific pathophysiological triggers associated with HIV-1 infection is also uncertain. Given these circumstances we hypothesized that HERV-K envelope might exhibit increased expression in the brain during HIV infection. We observed differential expression of the HERV-K(II) envelope in the brain depending on the host neural cell type and disease state. Moreover, HERV-K(II) Env expression in neuronal cells was protective during *in vitro* and *in vivo* exposure to cytotoxic HIV-1 circumstances.

## Results

### HERV expression in healthy human brain

**Figure 1:**
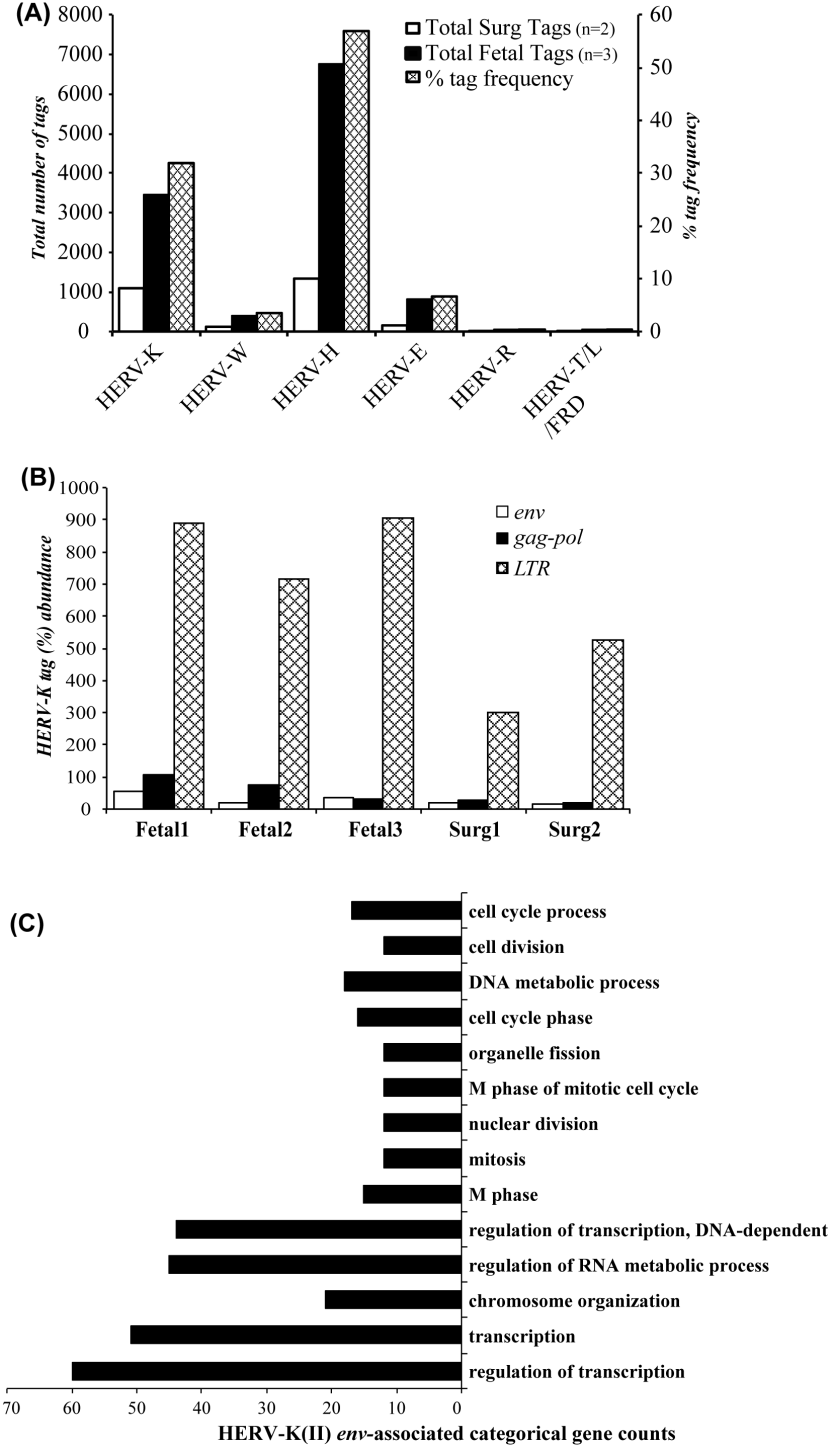
Deep sequencing analyses of HERVs in healthy brain. (**A**) Deep sequencing of the fetal and surgically resected (Surg) brain samples revealed that HERV-H exhibited the highest tag frequency and median number of tags followed by HERV-K. (**B**) When analyzing the HERV-K tags, LTR tags were most abundant, followed by *gag-pol* and then the *env* region tags (tags were normalized to respective gene lengths) (**C**) All host genes with transcript expression profiles correlated with HERV-K(II) *env* tag abundance (r^2^ **≥**0.5) were analyzed using the DAVID tools [58] for enriched gene ontology (GO) terms. Genes related to cell cycle functions and chromosomal organization were most strongly associated with HERV-K(II) *env* expression. With the use of DAVID bioinformatics resources [59], the predicted target genes were classified according to KEGG functional annotations to identify pathways that were actively regulated by HERV-K(II) *env* transcripts in brain tissue. The most over-represented GO term belonged to the transcriptional regulation and chromosome organization followed by different stages of cell cycle pathway. (Mann Whitney t test, *p<0.05, **p<0.01).

Although HERVs have been shown to be expressed in the human brain [20], their comparative expression levels have not been assessed to date using unbiased tools such as deep sequencing. The median number of HERV tags generated from human fetal (n = 3) and surgically resected (n = 2) brain RNA was 2738 tags per patient specimen by deep sequencing transcriptomic analysis while 31% belonged to the HERV-K family, which was only exceeded by the HERV-H family (57%) (**Figure 1A**). However, sequence tags were also assignable to HERV-W, -R, -E and –FRD. Fetal brains exhibited higher levels of HERV sequences for all HERV families (**Figure 1B**). Further analysis of the HERV-K tags revealed that LTR sequences were the most frequently detected tags among all patient brain specimens although *pol-gag* and *env* sequences were also detected (**Figure 1B**). LTR sequences dominated the total tag counts in all HERV families; for example, HERV-H, which contained the highest percentage tag frequency, displayed median tag frequencies of 0.5%, 17.6% and 81.8% for *env*, *gag-pol* and LTR, respectively. All host genes with transcript expression profiles were correlated with HERV-K(II) *env* tag abundance in the corresponding sample; based on sequence and bioinformatic analyses of differentially expressed host genes, there was substantial enrichment of host transcription-, translation- and cell cycle-related mRNAs associated with HERV-K(II) tag abundance (**Figure 1C**). Of interest, HERV-K *env* sequences are located throughout the human genome (**Figure S1A**) although the density of HERV-K LTR sequences was overall highest in specific chromosomes (**Figure S1B**). These findings highlighted the diverse expression of HERV genes in the human brain together with showing age-related expression and associations with fundamental host gene functions.

### *Ex vivo* modulation of HERV-K(II) env expression

**Figure 2:**
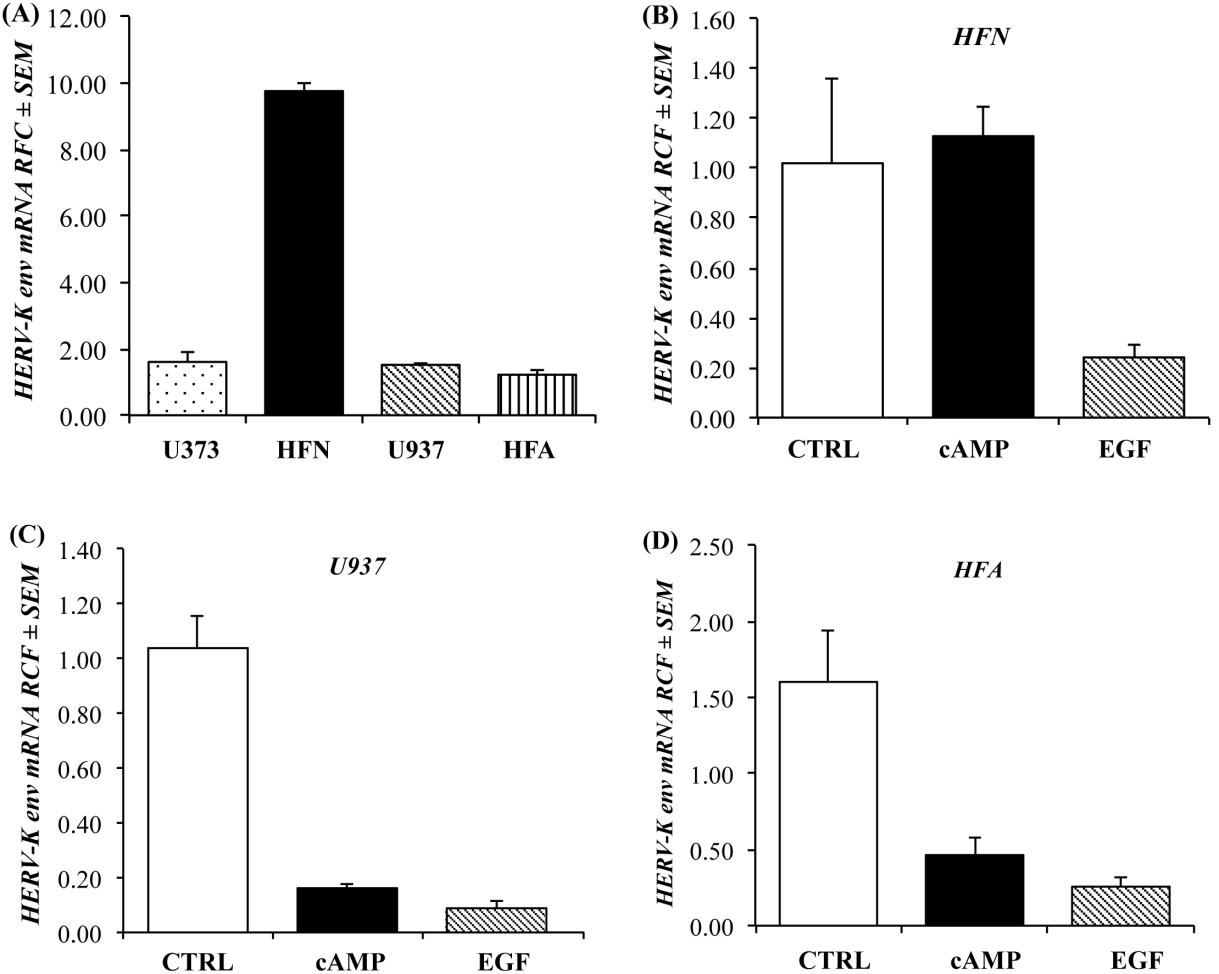
Activation of HERV-K(II) *env* by cAMP and EGF in different human cell lines (**A**) Individual cell lines displayed differential constitutive HERV-K(II) *env* expression profiles. (**B**) Upon treatment of human fetal neurons, db-cAMP did not have any effects on HERV-K(II) *env* expression but EGF down-regulated HERV-K(II) expression. (**C**) U937 and (**D**) HFA showed decreased in HERV-K(II) *env* expression upon both db-cAMP and EGF exposure. (n = 4 replicates per group across two independent experiments).

Stimulation of trophoblast cell fusion and differentiation by cyclic AMP (cAMP) has been associated with increased HERV-W *env* transcript and protein expression [27]. We investigated the effects of cAMP and epidermal growth factor (EGF) exposure on the expression profile of HERV-K *env* transcripts in different cell types. In this assay, HERV-K(II) *env* transcript levels were measured in U373 (human astrocytoma cell line), HFN (human fetal neurons), U937 (human leukemic monocyte lymphoma cell line) and HFA (human fetal astrocytes). HFNs showed the highest constitutive levels of HERV-K(II) *env* transcripts among all cell lines (**Figure 2A**). There was no effect of cAMP exposure on HERV-K(II) *env* transcript levels in HFNs, whereas there was decrease in HERV-K(II) *env* transcription in EGF-exposed HFNs (**Figure 2B**). Both cAMP- and EGF-exposed U937 (**Figure 2C**) and HFA (**Figure 2D**) cells showed a reduction in HERV-K(II) *env* transcripts. These observations highlighted HERV-K(II) *env* expression was differentially regulated depending on the individual cell type and stimulus.

### HERV-K(II) Env induces neurotrophin expression and neuronal process growth

**Figure 3:**
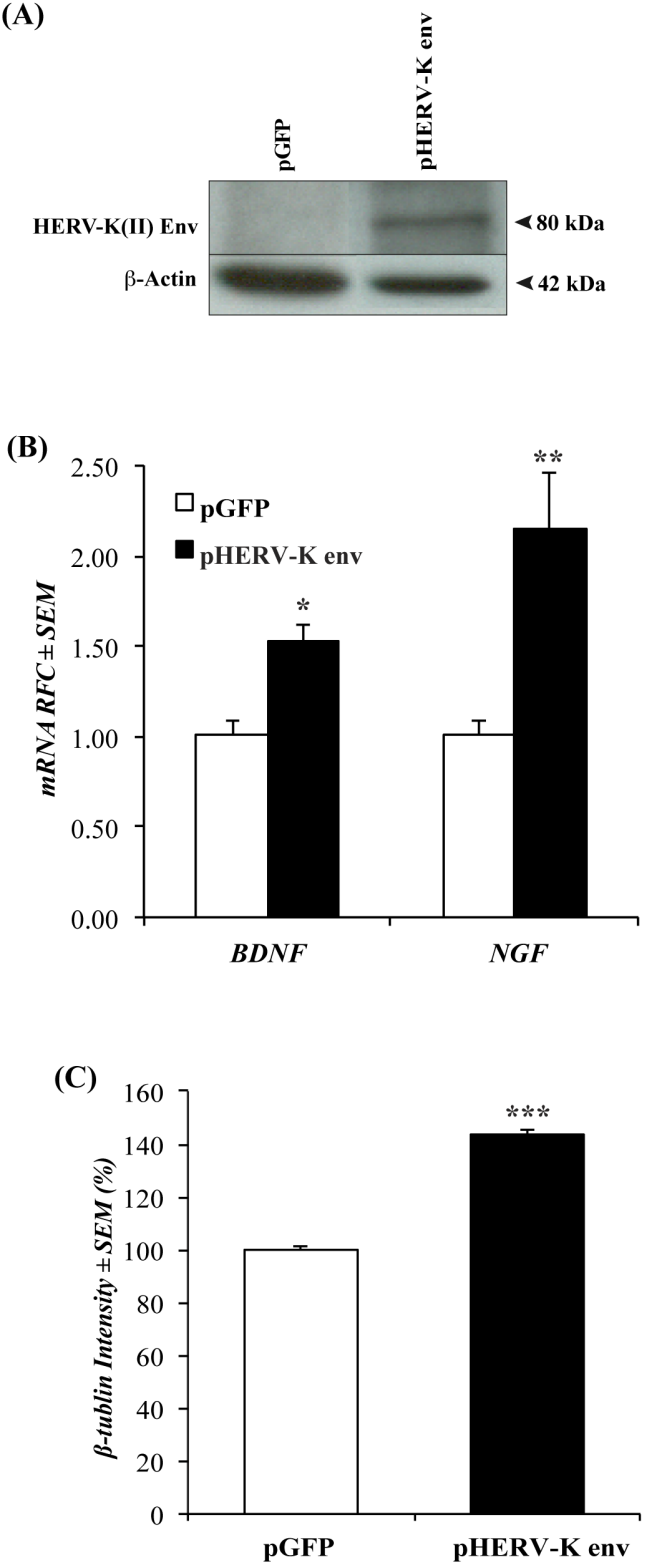
Over expression of HERV-K(II) Env exerts neurotrophic effects: (**A**) Transfection of the pHERV-Kenv plasmid into SK-N-SH cells showed HERV-K(II) Env immunoreactivity at the predicted molecular weight on western blot. (**B**) Upon treatment with supernatants from SK-N-SH cells transfected with pHERV-Kenv plasmid, HFN showed increases in *BDNF* and *NGF* transcript abundance compared to the control vector transfected cells. (n = 3, with technical quadruplicates) (**C**) βIII-tubulin expression in HFN following 24-hour exposure to supernatants from HFA-transfected with the pHERV-Kenv or the control vector, showing an increase in βIII-tubulin immunoreactivity in cells exposed to HERV-K Env-transfected cells. (n = 2, with technical octuplicates) (Student t test, *p<0.05, **p<0.01).

Transfection of the human neuronal cell line, SK-N-SH, with an HERV-K(II) *env-*encoding vector (pHERV-Kenv) disclosed HERV-K(II) Env immunoreactivity at the predicted molecular weight (80 kDa) although HERV-K(II) immunoreactivity was not detected in cells transfected with a control vector (pGFP) (**Figure 3A**). In addition, transcripts encoded by *BDNF* and *NGF* were induced in HERV-K(II)-transfected SK-N-SH cells (**Figure 3B**) relative to cells transfected with the pGFP control vector. In addition, analyses of SK-N-SH cells transfected with the HERV-K(II) *env* containing vector showed increased levels of βIII-tubulin immunoreactivity compared to the control vector-transfected cells (**Figure 3C**). These findings implied that HERV-K(II) Env expression could be increased in human cells and might confer neurotrophic effects on neuronal cells.

### HERV-K(II) Env expression in human brain during HIV/AIDS

**Figure 4:**
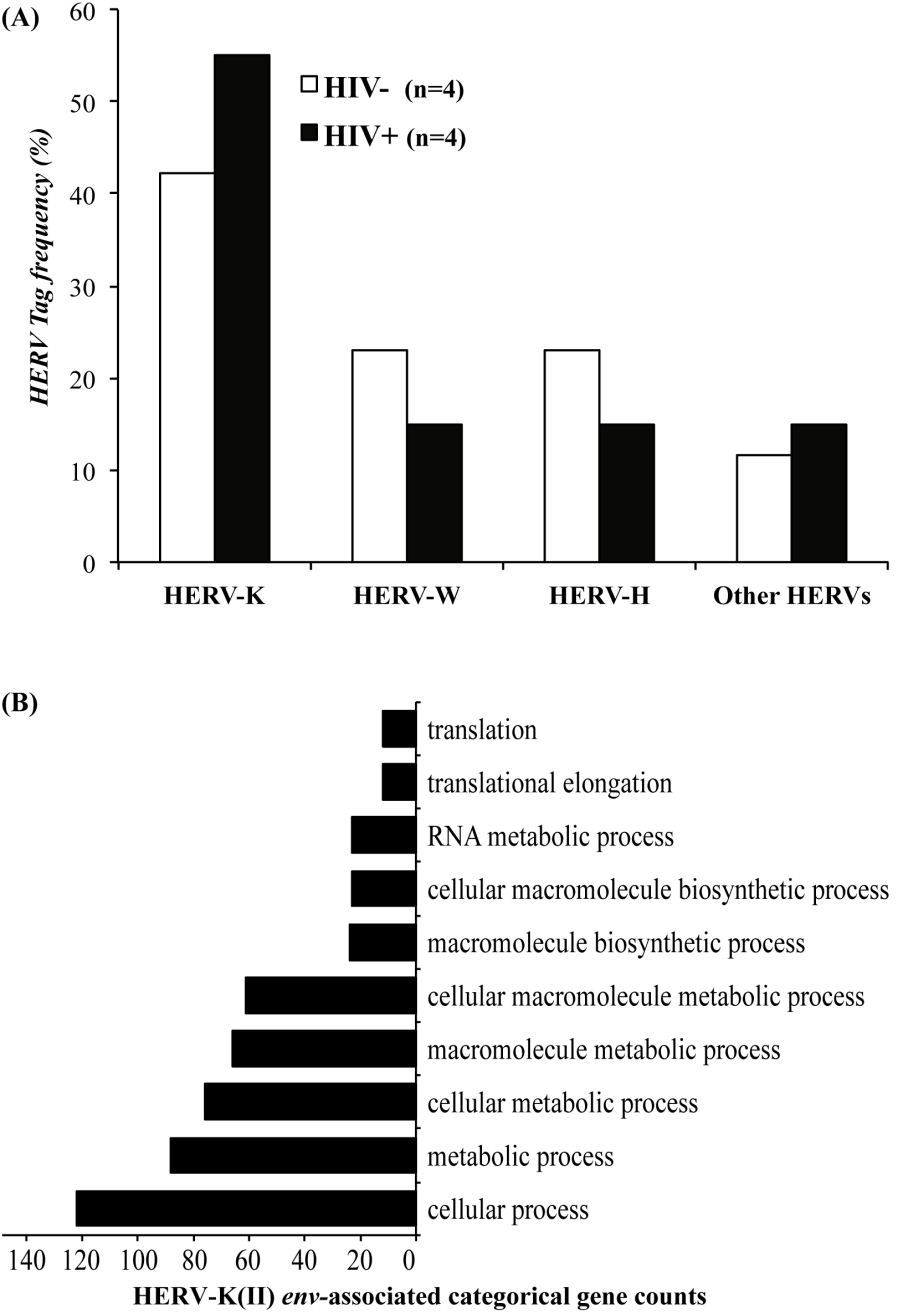
HERV transcripts in HIV− infected brain specimens. (**A**) Deep sequencing of the HIV− and HIV+ autopsied cerebral white matter revealed a higher tag frequency of HERV-K in both clinical groups compared to other HERVs. (**B**) With the use of the DAVID bioinformatics resources, the predicted target genes were classified according to KEGG functional annotations to identify pathways that were actively regulated by HERV-K(II) *env* transcripts in brain tissue.

Previous studies have suggested that HERV-K(II) *pol* transcripts are expressed in the brain in disease [11], [20]. To investigate the *in vivo* abundance and specificity of HERV expression, we analyzed cerebral white matter from patients with HIV/AIDS (HIV+, n = 3) and uninfected persons with other diseases as controls (HIV−, n = 3) by transcriptomic deep sequencing. Massively parallel sequencing of samples produced a large number of short reads/tags, which were assembled into contigs (overlap length of 36–77 nucleotides). Overall 5,640,659–8,803,479 tags were obtained depending upon the individual sample, of these, 32.8% were mapped to human rRNA (one or more read per pair), 12.3% were aligned to human transcriptome, 34.0% to the human nuclear genome (but not to human transcriptome), 8.0% to human mitochondrion DNA (mtDNA), 0.1% to bacterial and viral sequences (but not to human genome or transcriptome) and 15% sequences were not found in sequence database queried. These studies revealed that the median HERV tag number specimen was 666 tags/specimen, of which 74% belonged to the HERV-K family; HERV-K tags were the most abundant tags detected in both clinical groups with the HIV+ specimens showing a higher HERV-K tag frequency than the HIV− group but HERV-W and HERV-H associated tags were more abundant in the HIV− group (**Figure 4A**). Sequence tags belonging to the HERV-W, -H and other HERVs were present in all specimens examined but the HIV HIV− group showed greater expression of these latter HERVs. Analysis of the relative abundance of all HERV-K *env* sequences tags showed no difference in tag numbers between the clinical groups. Comparison of the relative expression of different host genes in the HIV+ and HIV− groups’ brains disclosed that tags of multiple groups of host genes implicated in a wide range of fundamental functions were enriched in the HIV+ group’s brains based on gene ontology (GO) analyses (**Figure 4B**). These findings implied that there was differential expression of both HERV and host genes in the HIV+ and HIV− brain specimens.

**Figure 5:**
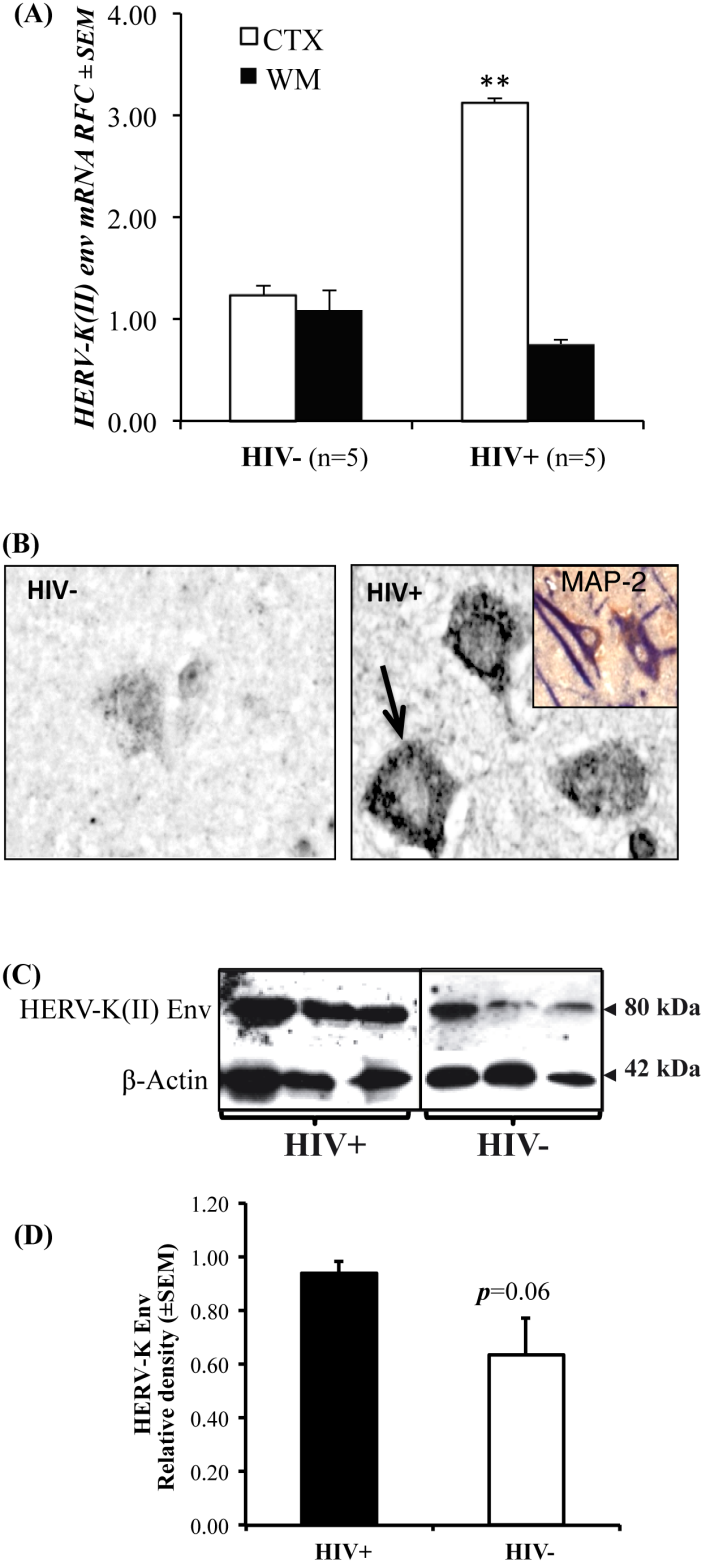
Brain expression of HERV-K(II) Env in HIV/AIDS: (**A**) HERV-K(II) *env* transcript analysis of HIV−) and HIV+ brains revealed high levels of HERV-K(II) *env* in cortex of HIV+ as compared to HIV− brains. (**B**) Immunohistochemical analyses of brain sections from HIV+ patients showed increased immunoreactivity of HERV-K(II) Env (arrow) protein in neurons as compared to the HIV− brain sections. HERV-K(II) Env protein expression co-localized in neurons expressing MAP-2 (insert: brown, MAP-2; blue, HERV-K Env). (**C**) In cerebral cortical specimens, HIV+ patients exhibited higher levels of HERV-K(II) Env detection than HIV− patients on immunoblotting of HERV-K(II) Env protein. (**D**) Quantitation of HERV-K(II) Env/β-actin band density on immunoblots (Original magnification: B-400X; insert, 200X). (Student t test, **p<0.01).

To extend these findings, we focused on HERV-K(II) Env expression, which showed a significant increase in HERV-K(II) *env* transcript levels in cerebral cortical specimens from HIV+ patients compared to matched white matter as well as cortex and white matter of HIV− patients using real time RT-PCR (**Figure 5A**). HERV-K(II) Env immunoreactivity in cerebral sections was minimal in HIV− patients (**Figure 5B**) but HIV+ brain sections displayed immunoreactivity in cells resembling cortical neurons, which was co-localized with MAP-2 immunoreactivity (**Figure 5B** **, insert**). Western blotting of cerebral cortex specimens from HIV− and HIV+ brains showed that HERV-K(II) Env expression was greater in the brains of HIV+ patients compared with HIV− patients (**Figure 5C**). Densitometry analyses of immunoblots showed that HERV-K Env expression was increased in the brains of HIV+ patients (**Figure 5D**). These findings suggested that HERV-K(II) Env was expressed in human cortical neurons, which was augmented during HIV infection.

### HERV-K(II) Env prevents neuronal injury

**Figure 6:**
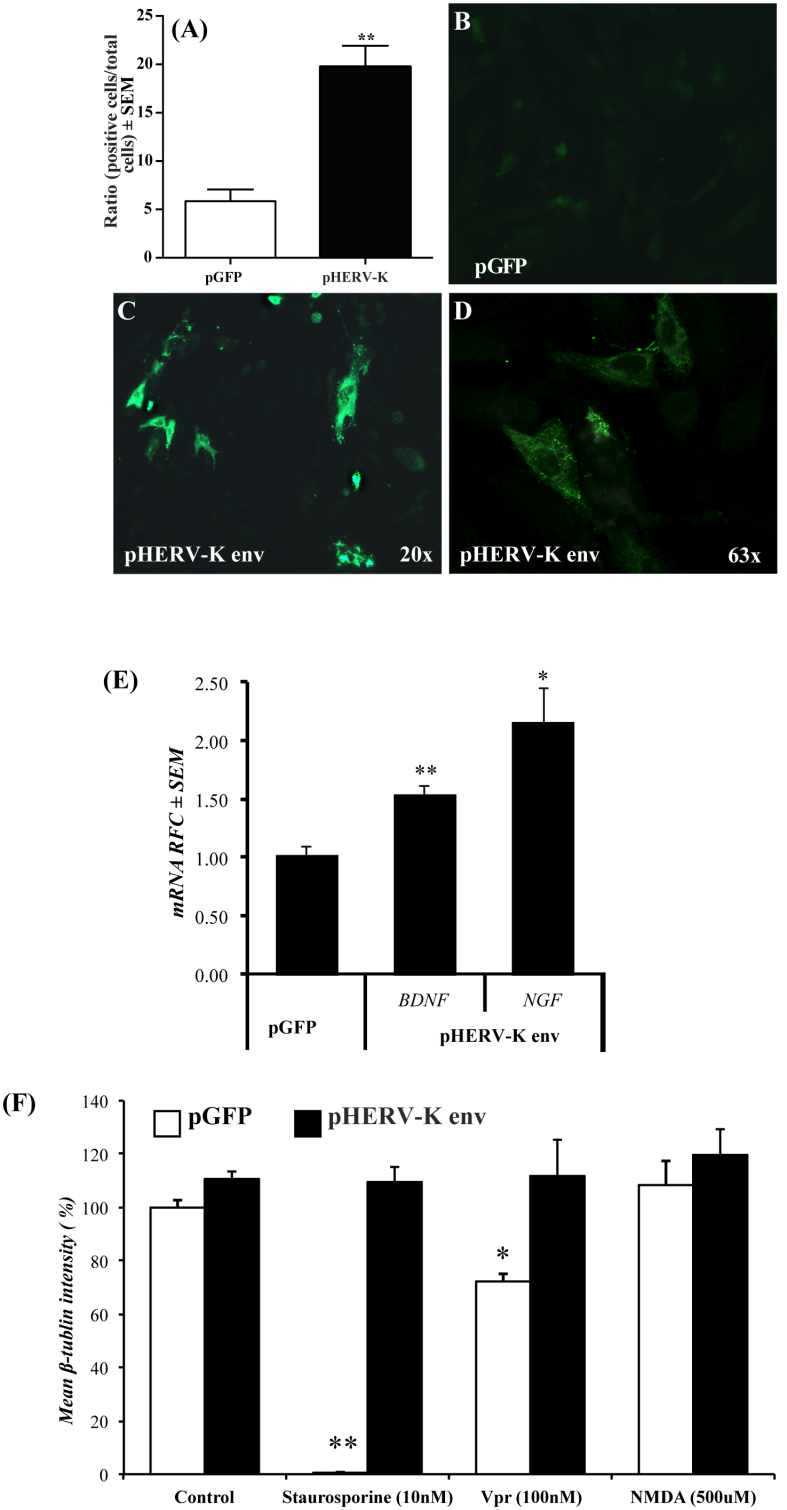
HERV-K(II) *env* transfection of neuronal cells was neuroprotective. (**A**) Analyses of SK-N-SH cells transfected with the pHERV-Kenv plasmid compared to the control (pGFP) showed that the efficiency of transfection was ∼20% (n = 3, with technical triplicates). (**B**) HERV-K(II) Env immunoreactivity was minimally detected in cells transfected with the control vector. pHERV-Kenv-transfected cells showed HERV-K(II) Env immunoreactivity at low (**C**) and high magnification (**D**). (**E**) Comparison of *BDNF* and *NGF* transcript levels in SKN-N-SH cells transfected with pGFP or pHERV-Kenv. (**F**) Exposure of pHERV-Kenv and control vector-transfected NG108 cells to staurosporine, HIV-1 Vpr or NMDA, showed that pHERV-Kenv-transfected cells were differentially protected depending on the neurotoxin. (Student t test, *p<0.05, ***p<0.001).

As both BDNF and NGF are known to exert neurotrophic actions and were induced by HERV-K(II) Env over-expression in neuronal cells (**Figure 3B**), we investigated the contributions of HERV-K(II) Env to neuronal viability. Cell lines were transfected with pGFP or pHERV-Kenv and subsequently analysed for relative HERV-K(II) *env* transcript abundance, displaying variable expression depending on the individual cell line (**Figure S2A**). HERV-K(II) Env immunopositive cells were detected in ∼5% of SK-N-SH transfected with pGFP, which rose to ∼20% in pHERV-Kenv-transfected cells (**Figure 6A**). Similarly, HERV-K(II) Env immunoreactivity in pGFP-transfected neuronal cells was minimally detected (**Figure 6B**) but exhibited robust immunoreactivity in HERV-K(II) *env*-transfected cells (**Figure 6C**) with cytosolic and plasma membrane immunoreactivity (**Figure 6D**). Transfection of the human neuronal cell line SK-N-SH with pHERV-Kenv resulted in increased transcript levels of *BDNF* and *NGF* transcripts compared to pGFP-transfected cells (**Figure 6E**). To evaluate cell viability with and without concurrent HERV-K(II) Env expression, the murine NG108 neuronal cell line was transfected with each vector and subsequently exposed to different neurotoxins (**Figure 6F**). Cell viability was found to be preserved differentially in the HERV-K(II) Env-transfected cells following exposure to staurosporine, the HIV-1 Vpr protein or NMDA relative to pGFP-transfected cells with ∼100% and ∼40% loss of pGFP-transfected cells following exposure to staurosporine and Vpr, respectively, relative to the pHERV-Kenv-transfected cells. These observations suggested that HERV-K(II) Env expression in neurons selectively prevented their injury upon exposure to different neurotoxic molecules.

### HERV-K(II) Env is neuroprotective *In Vivo*

**Figure 7:**
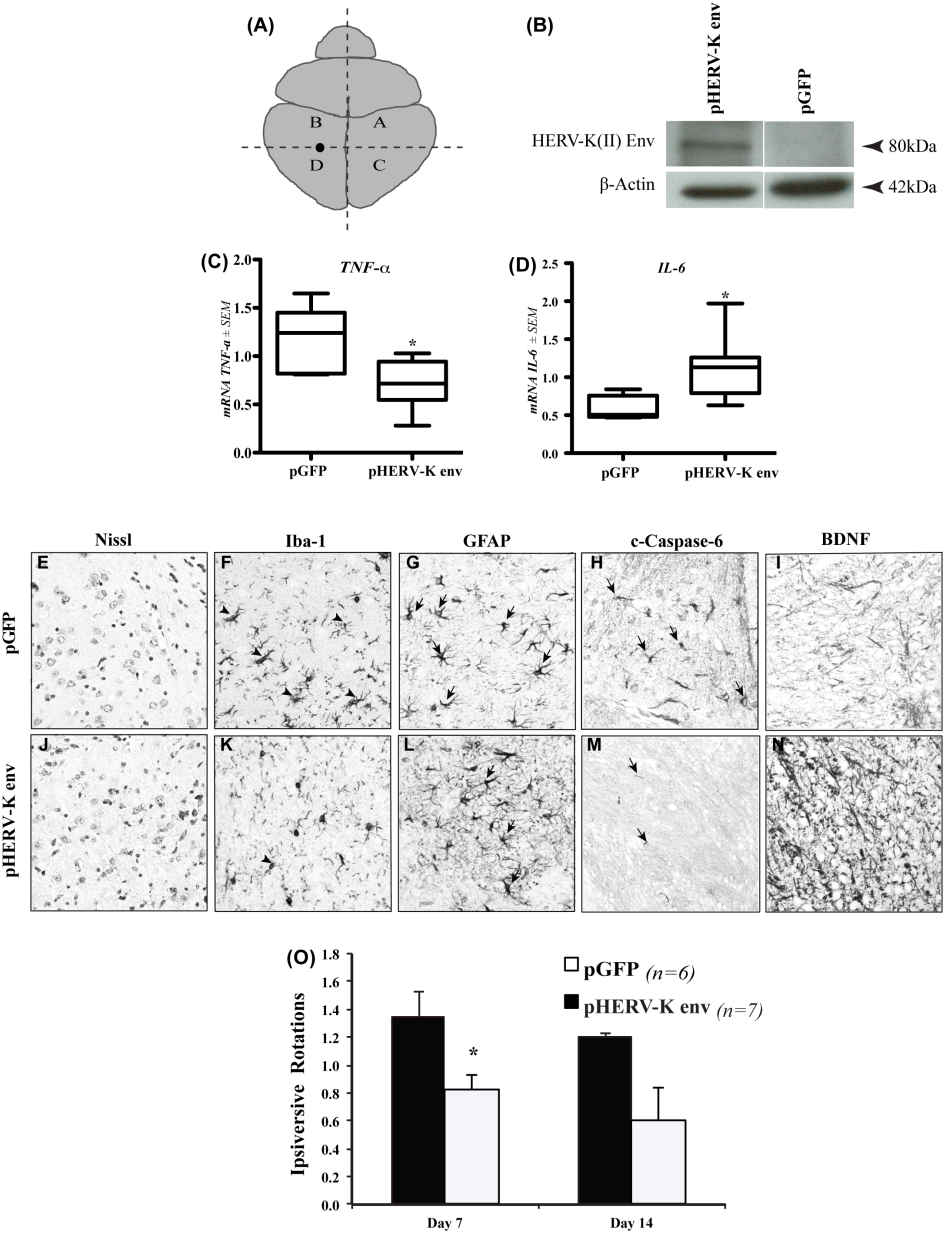
Neural stem cells expressing HERV-K(II) Env are protective in *vpr/RAG1^−/−^* animals. (**A**) Schematic of representation of C17.2 implantation site (marked by the •) in Vpr/RAG1^−/−^ mice. (**B**) Western blot showing HERV-K(II) Env immunoreactivity in transfected cells. (**C**) *TNF-α* expression was suppressed in the brains of animals implanted with cells expressing HERV-K(II) *env* while (**C**) *IL-6* was induced. Nissl staining showed similar striatal neuronal densities in animals implanted with cells transfected with either pGFP or pHERV-Kenv/pGFP (**E, I**). Immunohistochemistry revealed lower expression the microglia protein, Iba-1 (**K**) **and** higher expression levels astrocyte protein, GFAP (**L**) in HERV-K(II) *env* implanted brains compared to control vector (*pGFP*) implanted animals (**F, G**), respectively. Cleaved caspase-6 immunoreactvity was comparative reduced in striatum of animals receiving cells transfected with pHERV-Kenv/pGFP (**M**) but BDNF immunoreactivity was increased in the same animals (**N**) compared to controls (**H, I**). (**O**) At days 7 and 14, neurobehavioral deficits were greater in terms of ipsiversive rotations among the animals implanted with c17.2 cells transfected with the *pGFP* vector. (Original magnification: E–J, 400X) (Mann-Whitney test, *p<0.05).

Because of the apparent neuroprotective effects identified above, the *in vivo* effects of HERV-K(II) Env in HIV-1 *vpr/RAG1^−/−^* mice were investigated by stereotaxically implanting neural stem cells (NSCs) that were transfected with both the pHERV-Kenv and pGFP vectors or only the pGFP vector into the striatum (**Figure 7A**). Molecular, neuropathological and neurobehavioral studies were subsequently performed. Immunoblots of NSCs transfected with each of the above vectors revealed detection of HERV-K Env immunoreactivity in cells transfected with pHERV-Kenv/pGFP vectors (**Figure 7B**). Analyses of host transcript levels in the brains of *vpr/RAG1^−/−^* animals demonstrated that animals implanted with cells expressing HERV-K(II) *env* exhibited showed diminished transcript levels of *TNF-α* (**Figure 7C**), together with increased levels of *IL-6* (**Figure 7D**) as well as a trend towards increased *BDNF* expression in transgenic animals (**Figure S3D**). The *GFP* transgene transcript levels expressed by transfected cells were similar in animals receiving cells with the pGFP or the pHERV-Kenv/GFP vectors (**Figure S3C**). Neuropathological analyses revealed that Nissl-positive neurons in the striatum displayed similar morphology and density in animals implanted with NSCs transfected with pGFP (**Figure 7E**) and pHERV-Kenv/pGFP (**Figure 7J**). Iba-1 immunoreactivity was more abundant on hypertrophied cells, resembling microglia in the striatum of animals receiving the pGFP-transfected NSCs (**Figure 7F**) compared to animals receiving cells expressing HERV-K(II) Env (**Figure 7H**). GFAP immunoreactivity in astrocytes was increased in the animals receiving the pHERV-Kenv/pGFP-transfected cells (**Figure 7L**). In keeping with previous studies from our group, cleaved caspase-6 immunoreactivity was increased on cells resembling astrocytes in the striatum of animals implanted with NSCs transfected with pGFP (**Figure 7H**) compared to those implanted with NSCs expressing HERV-K(II) env (**Figure 7M**). In contrast, BDNF immunostaining was abundant the striatum of animals implanted with NSCs expressing HERV-K(II) Env (**Figure 7M**) compared to those implanted with NSCs transfected with pGFP alone (**Figure 7I**).

In neurobehavioral studies, animals that received the pGFP vector-transfected cells exhibited greater neurobehavioral deficits in terms of rotary behavior at days 7 and 14 post-striatal implantation of transfected cells compared to animals receiving cells transfected with the pHERV-Kenv/pGFP vectors (**Figure 7K**), underscoring the potential neuroprotective properties exerted by HERV-K(II) Env.

## Discussion

The present studies represent the first unbiased analysis of HERV transcript abundance in the human brain in both health and disease. HERV-K was among the most abundant HERVs identified; not surprisingly, the HERV-K LTR was the most frequently detected viral sequence. However, the HERV-K(II) Env was also observed in all brain specimens with the highest expression in neurons. Moreover, its expression was increased in the setting of HIV/AIDS in terms of both transcript and protein levels. Overexpression of HERV-K(II) Env in human neuronal cells induced neurotrophin expression and ensuing neuronal process extension while its *in vivo* expression in neural stem cells exerted beneficial effects in terms of reduced neuroinflammation (diminished microglial activation and *TNFα* expression) and improved neurobehavioral outcomes. Collectively, these observations point to a capacity for HERV-K(II) Env expression to be cell-type specific but also to respond to pathogenic stimuli in a manner that enhanced host fitness through preserved brain function.

Chronic neurodegeneration during HIV-1 infection of the nervous system remains a major clinical challenge, manifested as HIV− associated neurocognitive disorders, despite the burgeoning availability of highly active antiretroviral therapies [28]. The principal mechanisms by which HIV-1 injures the brain is through the release of virus-encoded proteins (e.g., Vpr, Tat, Env) or induction and release of potential immunopathogenic host molecules (e.g., TNF-α) from infected or activated glial cells and leukocytes [29], [30]. These secreted factors are toxic to neurons depending on the proximal concentrations, duration of exposure and host susceptibility factors (i.e. age), leading to apoptosis or necrosis but are also able to accentuate inflammation within the brain. Indeed, the present Vpr transgenic mouse, which selectively expresses Vpr protein in myeloid cells, exhibits a robust neurodegenerative phenotype defined by synapto-dendritic and neuronal loss coupled with worsened neurobehavioral performance on tasks of executive and motor functions [31]. These pathogenic circumstances represent a perturbed biological environment within the brain and thus induction of host molecules, which could avert or restrict host injury, is a plausible (and desirable) response to HIV infection.

Induction of HERV-K expression during HIV/AIDS is a recognized phenomenon in blood and appears to be associated with disease progression during HIV/AIDS [32], [33], [34]. Conversely, HERV-K *pol* transcripts appear to be induced in central nervous system tissues from patients with amyotrophic lateral sclerosis with the reverse transcriptase protein principally localized in cortical neurons and associated with TDP-43 expression [11]. Similarly, we also observed HERV-K(II) Env expression chiefly in cortical neurons as well as in cultured human fetal neurons. Given that neurons are terminally differentiated cells, they require robust protective mechanisms to survive; the conserved ability to induce expression of an ancient virus-encoded protein could be a valuable evolutionary strategy. The ability of HERV-K(II) Env to mediate activation of the neurotrophins, BDNF and NGF, which are altered in HIV/AIDS implies HERV overexpression might have an intrinsic adaptive function by reducing the brain’s susceptibility to neuronal injury. As mentioned above, other HERVs have been shown to be overexpressed in neurological diseases within different cell types including glia. For example, the HERV-W Env protein, Syncytin-1, is highly expressed in astrocytes in the brains of multiple sclerosis (MS) patients [7], [35] [36], [37], [38] and mediates endoplasmic reticulum stress *in vitro* and *in vivo* in astrocytes [6]. In fact, Syncytin-1 overexpression in the central nervous system during MS is pathogenic, resulting in neuroinflammation with ensuing oligodendrocyte (but not neuronal) injury and death [6], [38], [39]. By contrast, the murine endogenous retroviral envelope proteins, Syncytin A and B, are not induced in animal models of MS (Power, *unpublished*) emphasizing the species-specificity and diversity of responses among different endogenous retroviruses.

While *in vivo* HERV-K induction in blood is a consistent feature of HIV/AIDS, its *in vitro* activation is more variable, perhaps reflecting the different cell types involved [40]. However, increased HERV-K expression in human cortical neurons was a constant feature in this study of HIV/AIDS as well as in a previous study of a neurodegenerative disease, amyotrophic lateral sclerosis [11]. Several mechanisms underlying the relative HERV-K(II) Env induction in neurons include local inflammation secondary to the primary disease process in which inflammatory molecules such as cytokines activate the HERV-K(II) LTR as suggested for other retroviruses. Alternatively, a loss of CpG methylation leading to increased provirus transcriptional activity might permit HERV-K(II) induction in the setting of neuronal de-differentiation or injury. An (secondary) opportunistic infection such as CMV might also activate retroviral gene expression, which frequently complicates HIV/AIDS, as suggested for other opportunistic infections [16], [41]. In the case of HIV/AIDS, while neurons are not productively infected, the secretion of the HIV-1 viral proteins, Vpr or Tat, by infected myeloid/microglial cells could trans-activate retroviral gene expression in nearby neurons. Nonetheless, whatever the process is by which HERV-K(II) Env expression is enhanced, the resulting effect was beneficial to host neurons in the present studies through the concurrent stimulation of neurotrophin expression and ensuing neuroprotective effects. Indeed, this neuroprotective phenomenon was particularly evident in murine neuronal cells following transfection of the pHERV-Kenv vector (**Figure 6F**), possibly through the improved efficiency of transfection of this cell type as well as the absence of any residual HERV-K(II) *env* expression, creating a dominant negative effect. While TNF-α is widely recognized as toxic factor acting on neurons through engagement of its p75 receptor, the actions of IL-6, which was induced by HERV-K(II) expression in the implanted brains of *vpr/RAG1^−/−^* mice, is less clear. IL-6 expression is induced in many pathological circumstances but its downstream effects are divergent with both pathogenic and protective actions [42], [43], [44], [45]. The current studies imply that conservation and expression of HERVs in specific organs or cells might contribute to host adaptation to pathogenic circumstances, which could be exploited as preventative or therapeutic strategies in the future.

## Conclusions

The present studies demonstrate that HERV-K(II) Env was highly expressed in human neurons *in vitro* and *in vivo*, but was also induced in neurons during HIV/AIDS. Moreover, HERV-K(II) Env exerted protective effects on neuronal cells. These findings indicate that HERV gene-encoded proteins potentially mediate beneficial actions in circumstances of pathophysiological stress. Advantageous effects to host functions might underlie the conservation of HERVs within the human genome over time.

## Materials and Methods

### Human brain samples

Adult human brain (frontal lobe) specimens were collected at the time of autopsy or at the time of surgical resection for epilepsy with consent from HIV-1 sero-negative (uninfected) or -positive (HIV/AIDS) patients and stored at −80°C. All HIV/AIDS (HIV+) patients were AIDS-defined, as described previously [7], [46], [47], [48]. Uninfected disease controls were comprised of different diseases (HIV−, sepsis, cancer, multiple sclerosis, stroke). Surgically-resected brain specimens were derived from patients undergoing surgery for removal of an epileptogenic focus; tissue specimens used herein were remote from the epileptogenic lesion. The use of brain tissues is part of an ongoing research study (Pro0002291) approved by the University of Alberta Human Research Ethics Board. Human brain fetal tissues were obtained from 15–19 week (elective) aborted fetuses with written consent approved by the University of Alberta Human Research Ethics Board (Biomedical-Pro00027660) from which neurons and astrocytes were prepared. The protocols for obtaining brain specimens comply with all federal and institutional guidelines with special respect for the confidentiality of the donor’s identity and collected with consent.

### Human fetal neural cell cultures

To establish human neuronal cultures, fetal brain tissues were collected and prepared on the same day; the meninges were removed, tissues mechanically minced and a single cell suspension was prepared by trituration through serological pipettes, followed by digestion for 30 min with 0.25% trypsin (Gibco, Burlington, ON) and 0.2 mg/mL DNase I (Roche Diagnostics, Mannheim, Germany) and passage through a 70 micron cell strainer (BD Biosciences, Mississauga, ON). Cells were washed 2 times with fresh medium, and plated in T-75 flasks coated with poly-L-ornithine (Sigma Aldrich, Oakville, ON) at 6–8×10^7^ cells/75 mm2 flask in MEM supplemented with 10% FBS (Gibco), 2 mM L-glutamine (Gibco), 1 mM sodium pyruvate (Gibco), 1X MEM non-essential amino acids (Gibco), 0.1% dextrose (Sigma Aldrich), 100 U/mL Penicillin (Gibco), 100 µg/mL Streptomycin (Gibco), 0.5 µg/mL amphotericin B (Gibco) and 20 µg/mL gentamicin (Gibco). Cultures of neurons were additionally supplemented with 25 µM cytosine arabinoside (Sigma Aldrich, Oakville, ON) to prevent astrocyte growth.

### Cell lines

Cell lines were obtained from the American Type Culture Collection (ATCC; www.atcc.org) and cultured according to standard mammalian tissue culture protocols and sterile techniques. Human neuroblastoma (SK-N-SH) and murine neuronal (NG108) cells were cultured as monolayer in Dulbecco’s Modified Eagle Medium (DMEM). All media was supplemented with 10% FBS/100 units/ml penicillin/100 µg/ml streptomycin/2 mM L-glutamine. The RPMI medium was also supplemented with 1 mM sodium pyruvate/10 mM HEPES buffer. All tissue culture media and supplements were obtained from Invitrogen. Human fetal neurons (HFN), human fetal astrocytes (HFAs) and U937 cells [49] were cultured in 6 well plates and exposed to di-butyl cAMP (50 µg/ml) or epidermal growth factor (EGF (10 µg/ml) for 4 days. Following exposure, total RNA was extracted and relative mRNA levels of the different genes of interest were measured by a semi-quantitative reverse transcription PCR assay [7].

### Neural cell transfection and implantation

Murine neural stem (C17.2) cells [50] and human or murine neuroblastoma (SK-N-SH or NG108, respectively) cells were grown in Dulbecco’s modified Eagle’s medium (DMEM) supplemented with 10% fetal calf serum, 5% horse serum, 2 mM glutamine, penicillin/streptomycin/fungizone (Invitrogen, 100x stock, 1/100 ml media) as previously described [51], [52] in 25 cm^2^ uncoated tissue culture flasks at 37°C. Half of the media was changed every 3–4 days and cells were split (1∶20) weekly except when the cells were prepared for the implantation. Cells were transfected with a control plasmid (pBUD-GFP, a gift from Dr. David Vergote, University of Alberta) encoding enhanced green fluorescent protein (eGFP) or a HERV-K(II) Env encoding vector (pHERV-Kenv) [15], and henceforth these vectors were termed pGFP and pHERV-Kenv. For co-transfection, cells were grown overnight in medium in 6 well plates before transfecting with pGFP and pHERV-Kenv using lipofectamine reagents (Invitrogen) according to manufacturer’s protocol. Selection of the positively transfected cells was performed over 3 months with puromycin (2.5 µg/ml) and Zeocin. At the time of implantation, near confluent undifferentiated cells were trypsinized with Trypsin-EDTA (0.05%), washed 2 times with phosphate- pHERV-Kenv buffered saline (PBS) and re-suspended in Hank’s Balanced Salt Solution (HBSS) at a final concentration of 2×10^5^ viable cells/µl [53].

### Neurobehavioral studies

*Vpr/RAG1^−/−^* mice were generated by crossing Vpr transgenic mice which expressed HIV-1 Vpr under the control of the *c-fms* (M-CSF receptor) promoter, directing transgene expression chiefly in monocytoid cells [42], were crossed with RAG1^−/−^ animals, as previously reported [31] and were used for the present *in vivo* studies (Research study AUP00000318 approved by the University of Alberta Animal Care and Use Committee for Health Sciences). Neurobehavioral deficits were assessed by the Ungerstadt assay [43], [44]. Animals (4 weeks, n = 6–7) were anesthetized with Ketamine/Xylazine, ocular ointment was applied to their eyes to prevent drying and placed in a stereotaxic frame. The heads were cleaned with 70% ethanol, skin incised at the midline and a small cranial burr hole was made with a dental drill bit on a pre-marked skull area. The coordinates of implantation were 3.5 mm posterior, 2.5 mm lateral and 3 mm deep relative to the bregma resulting in an implantation site within the striatum (**Figure 7A**). 5 µl of transfected-cell suspension (HERV-K(II) *env/eGFP* or control, *eGFP*) containing ∼1×10^6^ viable cells were stereotactically implanted into the right striatum of each animal over 5 minutes. The wound was closed with cyanoacrylate glue (Vectabond). Ipsiversive rotational behavior, which is indicative of neurological injury, was measured over 10 min after intraperitoneal injection of amphetamine (1 mg/kg) on days 4, 7, 14 and 21 following intrastriatal injection. Animals were sacrificed after 21 days followed by intracardial perfusion with saline, followed by PBS/4% paraformaldehyde. The brain was removed and the tissue anterior to the implantation site was frozen at−80°C while the posterior tissue was post-fixed in PBS/4% paraformaldehyde embedded in paraffin from which 10-µm sections were prepared for immunohistochemical analysis.

### Neurotoxicity assays

HFN cells were cultured in 96-well flat bottom plates and exposed to either supernatants from SKN-N-SH cells transfected with plasmids encoding HERV-K(II) *env* (pHERV-Kenv) protein or control (pGFP). 48 h after treatment, cells were fixed, permeabilized and stained with mouse anti-β-tubulin antibody (1∶800 dilution, Sigma-Aldrich) as previously described [54]. Neuronal injury was quantified by βIII-tubulin immunoreactivity using Odyssey Imager (LI-COR, Lincoln, NE). Diminished βIII-tubulin immunoreactivity was indicative of reduced cellular viability [55]. For assaying the cytotoxic effects of different neurotoxins, murine neuronal NG108 cells were stably co-transfected with pBUD-GFP or pHERV-Kenv plasmids. The cells were grown on 4 well chamber slides to ∼60% confluency and then exposed to staurosporine (10 mg/ml), HIV-1 Vpr (100 nM) or NMDA (500 nM) for 24 hours [55], [56]. After the incubation period, cells were fixed with 4% formalin, washed in PBS containing 0.1% Triton X-100 (Sigma-Aldrich), and blocked for 90 min at 4°C with LI-COR Odyssey Blocking Buffer (LI-COR, Lincoln, NE), following which antibodies to βIII-tubulin (1∶1000) were applied to each well overnight and washed X3. A labeled secondary anti-mouse IgG antibody was applied for 1 hr, washed and then the relative immunoreactivity was assessed in each well [49], [55].

### Real-time RT-PCR

First-strand cDNA was synthesized by using aliquots of 1 µg of total RNA from cortex and basal ganglia, reverse transcriptase and random primers [7]. Specific genes were quantified by real-time PCR using i-Cycler MYIQ system (Bio-Rad, Mississauga, ON). cDNA prepared from total RNA of cultured cells was diluted 1∶1 with sterile water and 5 µl were thereafter used per RT-PCR reaction. Semi-quantitative analysis was performed by monitoring in real time the increase of fluorescence of the SYBR Green dye on a Bio-Rad detection system, as previously reported [57] and expressed as relative fold change (RFC) compared to control. Oligonucleotide primers are provided in Table 1.

**Table 1:** Oligonucleotide primers used in Real-time RT PCR analyses.

Primer name	Sequence (5′ → 3′)	T_a_ (°C)	Species
*GAPDH* forward	AGCCTTCTCCATGGTGGTGAAGAC	60	Human/Mouse
*GAPDH* reverse	CGGAGTCAACGGATTTGGTCG		
*HERV-K (II)* forward	CCTGCAGTCCAAAATTGGTT	55	Human
*HERV-K (II)*reverse	GGGGCAAGTTTTTCCCTTTAG		
*hIL-6* forward	ACCCCTGACCCAACCACAAAT	58	Human
*hIL-6* reverse	AGCTGCGCAGAATGAGATGAG		
*hTNF*α forward	CCCAGGGACCTCTCTCTAATCA	57	Human
*hTNF*α reverse	GCTACAGGCTTGTCACTCGG		
*hIFN-β* forward	CAGCAATTTTCAGTGTCAGAAGCT	57	Human
*hIFN-β* reverse	TCATCCTGTCCTTGAGGCAGTA		
*hGFAP* forward	GGACATCGAGATCGCCACCTACAG	60	Human
*hGFAP* reverse	CTCACCATCCCGCATCTCCACAGT		
*hIL-1*β forward	CCAAAGAAGAAGATGGAAAAGC	55	Human
*hIL-1*β reverse	GGTGCTGATGTACCAGTTGGG		
*hBDNF* forward	GAAAGTCCCGGTATCCAAAG	50	Human
*hBDNF* reverse	CCAGCCAATTCTCTTTTT		
*hNGF* forward	CCAAGGGAGCAGCTTTCTATCCTGG	60	Human
*hNGF* reverse	GGCAGTGTCAAGGGAATGCGAAGTT		
*GFP* forward	CCACAACATCGAGGACGGCA	55	pBud-GFP plasmid
*GFP* reverse	CGGGATCACTCTCGGCATGG		
*mTNF*α forward	ATGCTGGGACAGTGACCTGG	54	Mouse
*mTNF*α reverse	CCTTGATGGTGGTGCATGAG		
*mIL-6* forward	ATGGATGCTACCAAACTGGAT	54	Mouse
*mIL-6* reverse	TGAAGGACTCTGGCTTTGTCT		
*mIFN-β* forward	AAGAGTTACACTGCCTTTGCCATC	55	Mouse
*mIFN-β* reverse	CACTGTCTGCTGGTGGAGTTCATC		
*mGFAP* forward	GGACATCGAGATCGCCACCTACAG	55	Mouse
*mGFAP* reverse	CTCACCATCCCGCATCTCCACAGT		
*mBDNF* forward	AGTTCCACCAGGTGAGAAGA	55	Mouse
*mBDNF* reverse	GGTAATTTTTGTATTCCTCCAGCAGA		

### Immunohistochemistry

Formalin-fixed, paraffin-embedded sections of human brain tissue (frontal lobe) on glass slides were de-paraffinized and rehydrated using decreasing concentrations of ethanol. 4% PBS-buffered paraformaldehyde fixed mouse brains (left hemisphere) were embedded in paraffin and sections (5.0 µm thick) were cut with microtome onto glass slides (Reichert, Austria). The section slides were de-paraffinized in 2 changes of xylene for 5 minutes each followed by rehydration using a series of graded alcohols. Antigen retrieval was performed by boiling the slides in 0.01 M tri-sodium citrate buffer, pH 6, for 10 min followed by incubation with Levamisole to block endogenous alkaline phosphatase. Sections were then pre-incubated with 10% normal goat serum, 0.2% Triton X-100 overnight at 4°C to block nonspecific binding. To detect Iba-1, cleaved caspase-6, BDNF or GFAP immunoreactivity, slides were incubated overnight at 4°C with antibodies to MAP-2 (1∶800; Sigma, USA), Iba-1 (1∶1000, Waco), GFAP (1∶5000, DAKO), BDNF (1∶1000, eBioscience) and cleaved caspase-6 (1∶500, gift from Dr. Andrea LeBlanc, McGill University), diluted in 5% normal goat serum, 0.2% Triton X-100. Mouse brain sections were also Nissl stained. A secondary alkaline phosphatase–conjugated goat anti–mouse or anti-mouse antibody (Jackson ImmunoResearch Laboratories) followed by NBT/BCIP substrate (Vector Laboratories) was used for single labeling. For double labeling with HERV-K(II) Env, human brain sections pretreated with 0.3% hydrogen peroxide to block endogenous peroxidases were incubated with rabbit polyclonal HERV-K(II) env antibody (1∶200; Novus, USA), followed by biotinylated goat anti–rabbit antibody by avidin–biotin–peroxidase complexes (Vector Laboratories), then 3,3′-diaminobenzidine tetrachloride (Vector Laboratories) was applied.

### Western blotting

Brain tissue or transfected cells were lysed with Laemmli buffer with 0.1% β-mercaptoethanol and boiled at 95°C for 10 minutes. Proteins from whole cell lysates were separated using polyacrylamide gel electrophoresis and protein fractions were transferred to a nitrocellulose membrane overnight (Bio-Rad, Mississauga, ON, CA). The membrane was blocked with 5% milk for 1 hour and labeled with monoclonal mouse anti-HERV-K(II) Env antibody (1∶200; Novus, USA), overnight at 4°. The immunolabeled membrane was then probed with secondary peroxidase-conjugated goat anti-rabbit IgG (1∶500: Jackson ImmunoResearch Laboratories, Inc., West Grove, PA, USA) for 2 hrs. Anti-βIII-actin antibodies were used to assess gel loading (1∶1000) (Santa Cruz Biotechnology, Inc., Dallas, TX, USA). Membranes were developed with Pierce ECL Western blotting substrate (Fisher Scientific, Ottawa, ON, Canada) and exposed on film (Canon Canada, Inc., Mississauga, ON, Canada).

### Deep sequencing and analyses of brain transcriptome

The high throughput brain transcriptome analysis was performed as described previously [7]. In brief ten micrograms of total RNA from fetal, surgical and clinical brain samples were used for the cDNA synthesis using ds-cDNA synthesis kit (Invitrogen) according to the manufacturer’s instructions. The resulting ds-cDNA was cleaned and single end tag (SET) sequencing was performed using the Illumina Genome Analyzer per the manufacturer’s instructions. Short read sequences (tags) obtained from the Illumina Genome Analyzer were mapped to the reference HERV mRNAs from the NCBI database (study accession number SRP032168).

### Bioinformatic analyses

The sequence tags derived from deep sequencing of healthy surgical and clinical brain samples were unambiguously assigned to different HERV families and host genes and were analyzed for the abundance of different HERVs and host genes. Bioinformatic analysis was performed to gain insight into the functional aspects of host genes with expression levels highly correlated with those of HERV-K(II) *env*. To account for differences in sequence tags obtained from different sets of experiments and variation in starting materials (cDNA) was reconciled by normalizing different genes across all the samples (global normalization), which assumes the distribution of gene expression over different experiments was similar. All genes, which passed the filtering criteria (≥2 tags detected) and showed a high degree of correlation with respect to HERV-K(II) *env* transcript levels (Pearson r^2^≥0.5) in each sample, were analyzed in context of the BP_FAT gene ontology (GO) terms for overrepresented functional classes and tissue specificity examination using the DAVID tool (http://david.abcc.ncifcrf.gov). For clinical samples GO analysis was performed on the genes passing the above criteria as well as showing > = 0.3 fold change compared to controls.

### Statistical analyses

Experimental variables were analyzed by Student t or Kruskal-Wallis tests for parametric or non-parametric continuous variables and the Chi-square test for categorical variables. *In vitro* data were tested by one-way ANOVA with Bonferroni *post hoc* tests. The level of significance was defined as *p*<0.05.

## Supporting Information

Figure S1 **(A) Human-specific HERV-K insertion loci in the human genome.** The red arrows indicated the chromosomal locations of truncated and full-length HERV-K elements i.e. Env, LTR and central ORFs. (B) A heatmap showing relative expression of different HERV-K elements on human genome.
(EPS)

Figure S2 Relative expression of HERV-K(II) *env* in *pHERV-Kenv* transfected HEK, SK-N-SH and C17.2 cell lines normalized to the control vector (*pGFP*) transfected-matched cell line.
(TIF)

Figure S3 **Transcript levels** ***vpr/RAG1^−/−^*** **mice implanted with HERV-K(II) env/eGFP expressing c17.2 cells.** Analyses of gene expression from implanted/non-implanted hemispheres of brain sections did not reveal significant differences in **(A)** *IFN-β,* **(B)** *GFAP*, **(C)** *GFP* and **(D)** *BDNF* transcript levels.
(TIF)
