# Supplementary figures and images for: Human Endogenous Retrovirus-K(II) Envelope Induction Protects Neurons during HIV/AIDS
Source: PLoS One. 2014 Jul 2;9(7):e97984. doi: 10.1371/journal.pone.0097984 (PMC4079299; doi:10.1371/journal.pone.0097984)

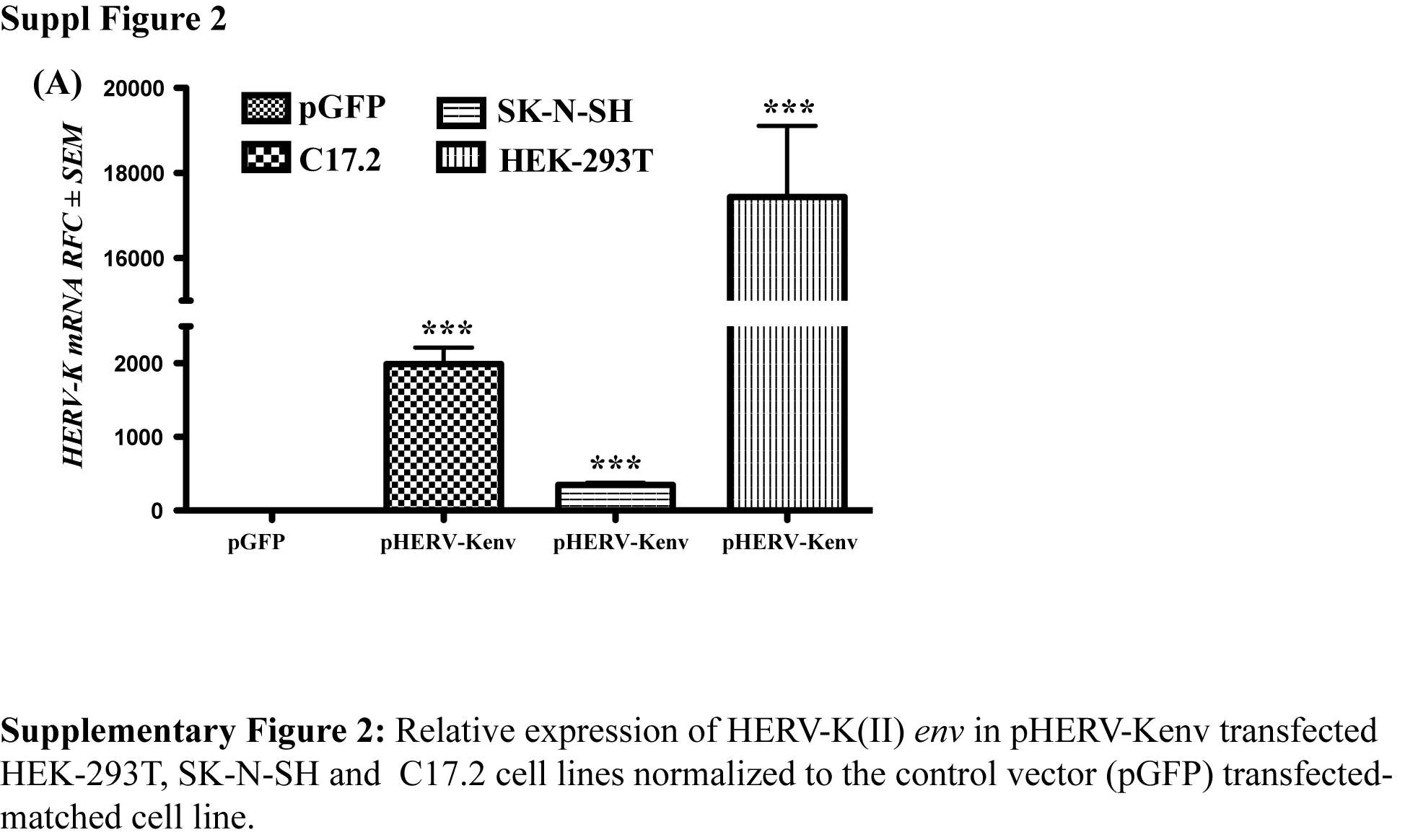

Supplement: Figure S2 — Relative expression of HERV-K(II) env in pHERV-Kenv transfected HEK, SK-N-SH and C17.2 cell lines normalized to the control vector (pGFP) transfected-matched cell line. (TIF) [file pone.0097984.s002.tif]

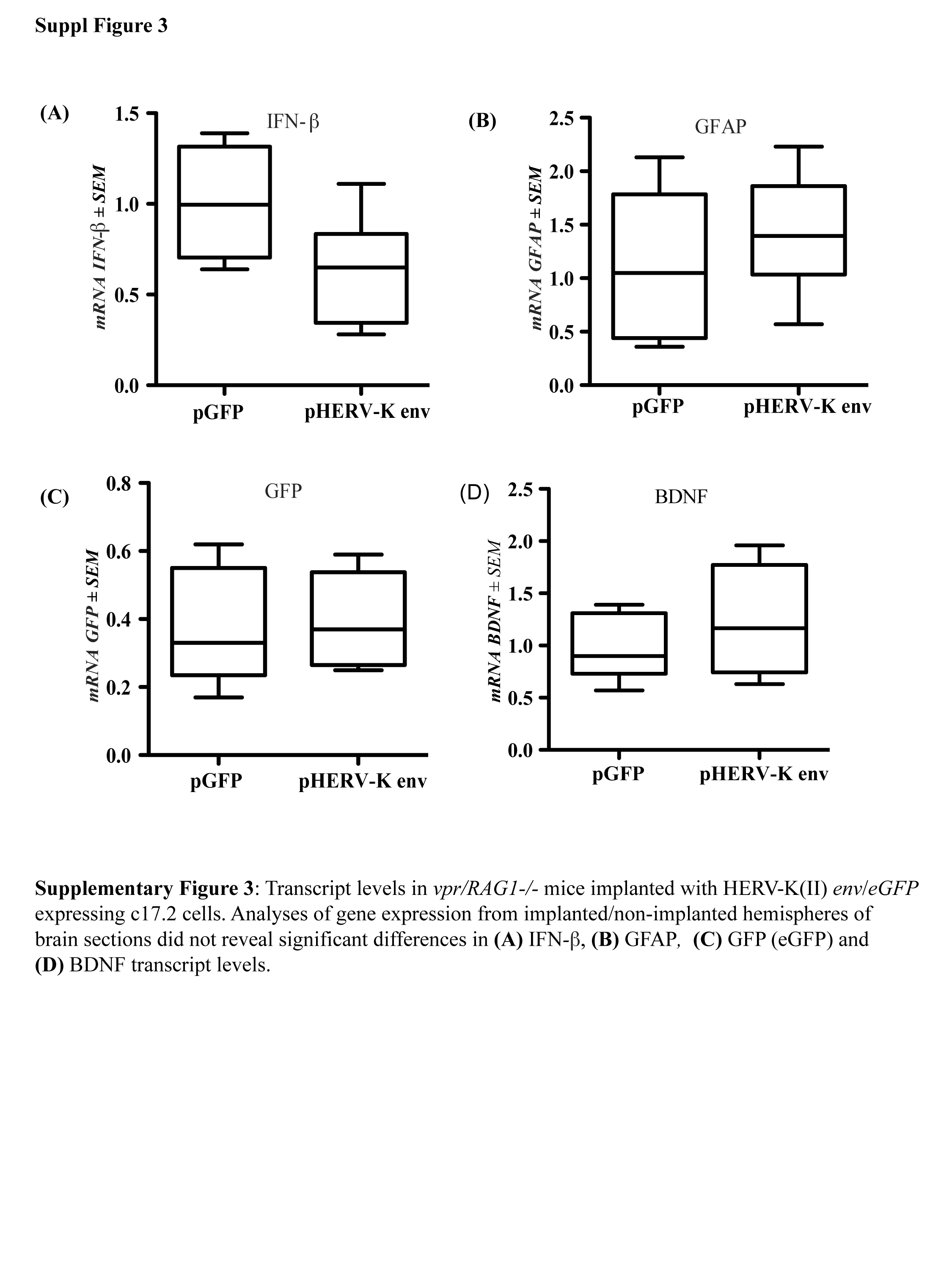

Supplement: Figure S3 — Transcript levels vpr/RAG1−/− mice implanted with HERV-K(II) env/eGFP expressing c17.2 cells. Analyses of gene expression from implanted/non-implanted hemispheres of brain sections did not reveal significant differences in (A) IFN-β, (B) GFAP, (C) GFP and (D) BDNF transcript levels. (TIF) [file pone.0097984.s003.tif]
